# Aspartame Sensitivity? A Double Blind Randomised Crossover Study

**DOI:** 10.1371/journal.pone.0116212

**Published:** 2015-03-18

**Authors:** Thozhukat Sathyapalan, Natalie J. Thatcher, Richard Hammersley, Alan S. Rigby, Alexandros Pechlivanis, Nigel J. Gooderham, Elaine Holmes, Carel W. le Roux, Stephen L. Atkin, Fraser Courts

**Affiliations:** 1 Academic Endocrinology, Diabetes and Metabolism, Hull York Medical School, University of Hull, Hull, United Kingdom; 2 Food Standards Agency, London, United Kingdom; 3 Department of Psychology, University of Hull, Hull, United Kingdom; 4 Department of Academic Cardiology, University of Hull, Hull, United Kingdom; 5 Faculty of Medicine, Imperial College, London, United Kingdom; 6 Diabetes Complications Research Centre, Conway Institute, University College Dublin, Belfield, Ireland; 7 Weill Cornell Medical College Qatar, Education City PO Box 24144, Doha, Qatar; 8 Institute of Food Research, Norwich Research Park, Norwich, Norfolk NR4 7UA, United Kingdom; Bielefeld Evangelical Hospital, GERMANY

## Abstract

**Background:**

Aspartame is a commonly used intense artificial sweetener, being approximately 200 times sweeter than sucrose. There have been concerns over aspartame since approval in the 1980s including a large anecdotal database reporting severe symptoms. The objective of this study was to compare the acute symptom effects of aspartame to a control preparation.

**Methods:**

This was a double-blind randomized cross over study conducted in a clinical research unit in United Kingdom. Forty-eight individual who has self reported sensitivity to aspartame were compared to 48 age and gender matched aspartame non-sensitive individuals. They were given aspartame (100mg)-containing or control snack bars randomly at least 7 days apart. The main outcome measures were acute effects of aspartame measured using repeated ratings of 14 symptoms, biochemistry and metabonomics.

**Results:**

Aspartame sensitive and non-sensitive participants differed psychologically at baseline in handling feelings and perceived stress. Sensitive participants had higher triglycerides (2.05 ± 1.44 vs. 1.26 ± 0.84mmol/L; p value 0.008) and lower HDL-C (1.16 ± 0.34 vs. 1.35 ± 0.54 mmol/L; p value 0.04), reflected in ^1^H NMR serum analysis that showed differences in the baseline lipid content between the two groups. Urine metabonomic studies showed no significant differences. None of the rated symptoms differed between aspartame and control bars, or between sensitive and control participants. However, aspartame sensitive participants rated more symptoms particularly in the first test session, whether this was placebo or control. Aspartame and control bars affected GLP-1, GIP, tyrosine and phenylalanine levels equally in both aspartame sensitive and non-sensitive subjects.

**Conclusion:**

Using a comprehensive battery of psychological tests, biochemistry and state of the art metabonomics there was no evidence of any acute adverse responses to aspartame. This independent study gives reassurance to both regulatory bodies and the public that acute ingestion of aspartame does not have any detectable psychological or metabolic effects in humans.

**Trial Registration:**

ISRCTN Registry ISRCTN39650237

## Introduction

Aspartame (L-aspartic acid and L-phenylalanine bonded as a methyl ester) is an intense artificial sweetener, being approximately 200 times sweeter than sucrose. It is classified as a non-nutritive sweetener due to its intense sweetness and the need for very small amounts to sweeten foods. First approved by the U.S Food and Drug Administration (FDA)[1] aspartame is widely used throughout the food and drinks industry; approximately 2000 tonnes are consumed annually in Europe alone[2].

However, controversy over the safety of aspartame has existed since its approval in the 1980s. There are numerous individuals and website organisations promoting a huge volume of anecdotal literature detailing consumer concerns[3] that include cancer, multiple sclerosis, blindness, seizures, memory loss, depression, anxiety, obesity, birth defects and death, with anxieties regarding both acute and chronic exposure[2,4–8]. However, this is not supported in the scientific literature. Limited studies on symptoms exist; one study in 13 psychiatric patients suggested increased depression[7], interpretation of studies on headaches is restricted by low numbers of participant and poor study design[6,8–11]. The weight of evidence from these studies suggests no effect on behavior and cognition [12–18].

Consumer apprehension led to a European Food Safety Authority (EFSA) review of aspartame[19] including the compilation of a database of the most commonly reported symptoms associated with aspartame consumption[20–23]. A metabolic basis for these symptoms was considered but few studies exist: in a study in patients with type 2 diabetes plasma glucose and insulin levels fell similarly for sucrose and aspartame sweetened meals even though calorifically they were different [24].

In a 2010 review EFSA’s National Experts[25] reaffirmed aspartame safety but recognised the need to determine aspartame related symptoms and their metabolic mechanisms. This randomised double blind controlled crossover study was undertaken in self reported aspartame sensitive individuals and matched control subjects, using tests for psychological behavior complemented with state of the art metabonomics to ascertain if potential acute aspartame related symptoms were reflected in the profile of hundreds of metabolites, as even small changes in nutritional or psychological balance can result in changes in pattern and clustering.

## Study Design

The protocol for this trial and supporting CONSORT checklist are available as supporting information; see S1 CONSORT Checklist and S1 Protocol. The study was approved specifically by the East Yorkshire & North Lincolnshire Research Ethics Committee (ref: 09/H1304/46; 30 June 2009). The participants provided informed written consent to participate in this study. The above ethics committee has approved the above consent process.

In this single-centre double blind crossover study individuals self-reporting aspartame sensitivity (AS) (n = 48) were compared to age and gender matched non-sensitive (NS) participants (n = 48) recruited by media advertisement (press advertisements, television and radio). Individuals volunteering were classified as “self reported aspartame sensitive” individuals if they reported suffering one or more symptoms on multiple occasions and as a consequence were actively avoiding consumption of any aspartame in their diet. Individuals with any allergies other than possible aspartame sensitivity were excluded, as was those taking any over-the-counter or prescription medications during the previous 3 months. The participants were reimbursed travel expenses for attending the study visits. Participants were recruited for a 12 month period from Dec 2012 to Dec 2013.

Aspartame (100mg, equivalent to that consumed in a can of diet soda and less than the Acceptable Daily Intake maximum (ADI) recommended by FDA of 40mg/kg body weight/day) was incorporated into cold pressed cereal bars (Campden BRI, United Kingdom). In a prior taste trial, 40 healthy controls could not distinguish between aspartame or control bars. Randomisation was performed by Campden BRI. The study was unblinded only after all of the analyses had been undertaken.

In a ‘within-between’ design; the independent factors being participant type (sensitive or control) and test (aspartame versus control bar) and a within-subjects factor of test order.

There is little published guidance on sample size for pilot studies. Hertzog found that sample sizes between ranging between10–80 per group met a variety of aims (e.g., problems of data collection strategies, answering methodical queries, estimating variability, planning a larger study). This was irrespective of statistical test. Our choice (48 per group) was at the upper end of this range in line with Hertzog’s recommendations for planning future trials [26].

In addition 25 participants with self-reported aspartame sensitivity and 25 matched control participants were recruited to look at nocebo effect where they were given two lots of placebo (second part). The nocebo effect refers to the negative consequences of an inert treatment [27]. The intention was to analyse the second part of the study only if any discriminating effect was observed between the two arms in the first part of the study. Since there were no discriminating effect in the first study, the biochemical and psychological part of the second study was not performed.

### Study Procedures

Following an 8 hour overnight fast participants were given the aspartame or control bar, and crossed over at least 1 week apart. Bars were consumed within 5 minutes. Standard questionnaires were used to assess psychological condition at baseline, and 14 symptoms were rated repeatedly over 4 hours after consuming bars *(see section “psychological data”)*. Blood samples were taken at baseline and at 4 hours: urine specimens were provided at baseline, at 4, 12 and 24 hours.

### Biochemical analysis

Fasting venous blood was collected into serum gel, EDTA plasma and fluoride oxalate tubes. Plasma glucose was measured using a Synchron LX 20 analyzer (Beckman-Coulter, High Wycombe, UK). Other samples were separated by centrifugation at 2000 g for 15 min at 4°C, and aliquots stored at −80°C. Total cholesterol, triglycerides and high-density lipoprotein cholesterol (HDL-C) levels were measured enzymatically using a Synchron LX20 analyzer (Beckman-Coulter, High Wycombe, UK). Low-density lipoprotein cholesterol (LDL-C) was calculated using the Friedewald equation. Serum insulin was assayed using a competitive chemiluminescent immunoassay performed on the manufacturer’s DPC Immulite 2000 analyzer (Euro/DPC, Llanberis, UK). The analytical sensitivity of the insulin assay was 2μU/ml, coefficient of variation 6%, and there was no stated cross-reactivity with proinsulin. Insulin resistance was calculated using the homeostasis model assessment (HOMA) method (HOMA-IR = (insulin x glucose)/22.5). Serum CRP was measured by the high-sensitivity method on a Beckman DXC analyser. Incretin hormones, glucagon-like peptide-1 (GLP-1) and glucose-dependent insulinotropic polypeptide (GIP) were measured using ELISA methods (Linco Research, Missouri, MO, US). Plasma L-Tyr and L-Phe were measured simultaneously by LC-MS. Precision was assessed over 5 determinations of 8 analyte concentrations (1, 5, 10, 50, 100, 200, 300, 400 μM), giving mean deviations of 3.2% and 3.7% for L-Tyr and L-Phe respectively.

### Psychological data

Baseline: Beck Depression Inventory (BDI); Hospital Anxiety and Depression Scale (HADS); Perceived Stress Severity Scale (PSS); life events in the past 12 months (SRRI); Toronto Alexithymia Scale (TAS-20); Whiteley-7 Somatisation Index; State Trait Anxiety Inventory (STAI). After consumption: 14 symptoms were rated ten times during each session (unmarked visual analogue scales 0–100): Headache; mood swings; hot or flushed; nausea; tiredness; dizziness; nasal congestion; visual problems; tingling; bloating; hunger; thirst; happiness; arousal. Fifteen participants did not complete any symptom ratings and they were excluded from the psychological analyses. The 10th set of ratings were discarded as 37 were incomplete. With rated mood and symptom data of this kind there is no theoretical or a priori way of combining symptoms, which makes it necessary to analyse each symptom separately over time, leading to a large number of analyses and increasing the risk of Type 1 error. This was appropriate here, because the study sought any evidence of aspartame symptoms, even if these would not be consequential in the general population. Eight participants left at least one symptom rating blank across an entire test session, these data were kept missing, leading to varying numbers of participants in each symptom analysis. Other missing data were interpolated by replacing the missing value with the highest value from +/- two ratings in time on the same item. Symptom ratings were then summed across the nine ratings, and a total symptom score for nine ratings of 14 items was also calculated.

### Metabonomic analysis

Metabonomic analysis of serum and urine utilised high throughput nuclear magnetic resonance (NMR) spectroscopy, and liquid chromatography coupled to mass spectrometry (UPLC-MS) lipid analysis followed by multivariate chemometric data analysis.

### NMR data Acquisition

A Bruker Avance III 600 MHz spectrometer (Bruker Analytische GmbH, Rheinstetten, Germany), employing an inverse detection probe of 5 mm width with z-gradients, at 300 K was used.

Urine analysis: 1D NOESY pulse sequence with presaturation (25 Hz) for water signal suppression collecting 128 scans in 64-k data points over a spectral width of 12019.23 Hz, using a relaxation delay of 2 s, an acquisition time of 6.83 s and a mixing time of 0.01 s. Serum samples: Carr-Purcell-Meiboom-Gill (CPMG) utilising a T2 relaxation time filter to suppress the broad lipoprotein signals and presaturation (25 Hz) for water signal suppression, collecting 128 scans in 64-k data points over a spectral width of 7194.2 Hz, using a total delay of 6.55 s between pulse cycles.

NMR data were visually inspected, imported in Matlab, phased, baseline corrected and referenced to TSP (urine) and glucose (serum) using in-house routines. Probabilistic quotient normalisation was used for both urine and serum. Unsupervised and supervised chemometric analyses were performed utilising SIMCA 13 (Umetrics) software[28]. Analysis was blinded to aspartame consumption and subject group (AS or NS).

### LCMS Lipidomics acquisition

A subset of samples from 12 randomly picked individuals from each coded group, were analysed for lipids in +ESI and -ESI. XCMS peak picking [29], median fold change normalization[30] was performed prior to chemometric analysis in SIMCA 13.

### Statistical analyses

Normality of the data was determined using the Kolmogorov-Smirnov test. Biochemical and clinical data compared participant types and treatments by independent and paired t-tests as appropriate. Baseline psychological measures were compared between participant types (AS/NS) and session order (aspartame then control or control then aspartame) using multivariate analysis of variance. To reduce the number of analyses, for each symptom ratings after consumption were summed across four hours to give a composite score on each symptom for each test session out of 900, plus a sum of all symptom ratings. Before analysis of variance, all composite scores were log transformed to increase normality of their frequency distributions. Composite scores were then analysed by 15 repeated measures analyses of variance with session (first or second) as a repeated measures variable, and type of participant (AS/NS) and order of test as independent factors. In this design effects of aspartame would appear as a significant interaction between session and order, and significant effects of sensitivity by an interaction between session, order and type. Post-hoc comparisons for variables where there were significant effects were conducted using Mann-Whitney U tests. For all analyses, a two-tailed P ≤0.05 was considered to indicate statistical significance, correcting for 15 multiple comparisons would have given p<0.0033. For brevity, only significant and marginal results where p<0.06 are reported. All omitted effects were non-significant.

## Results

### Characteristics of the participants

**Fig 1:**
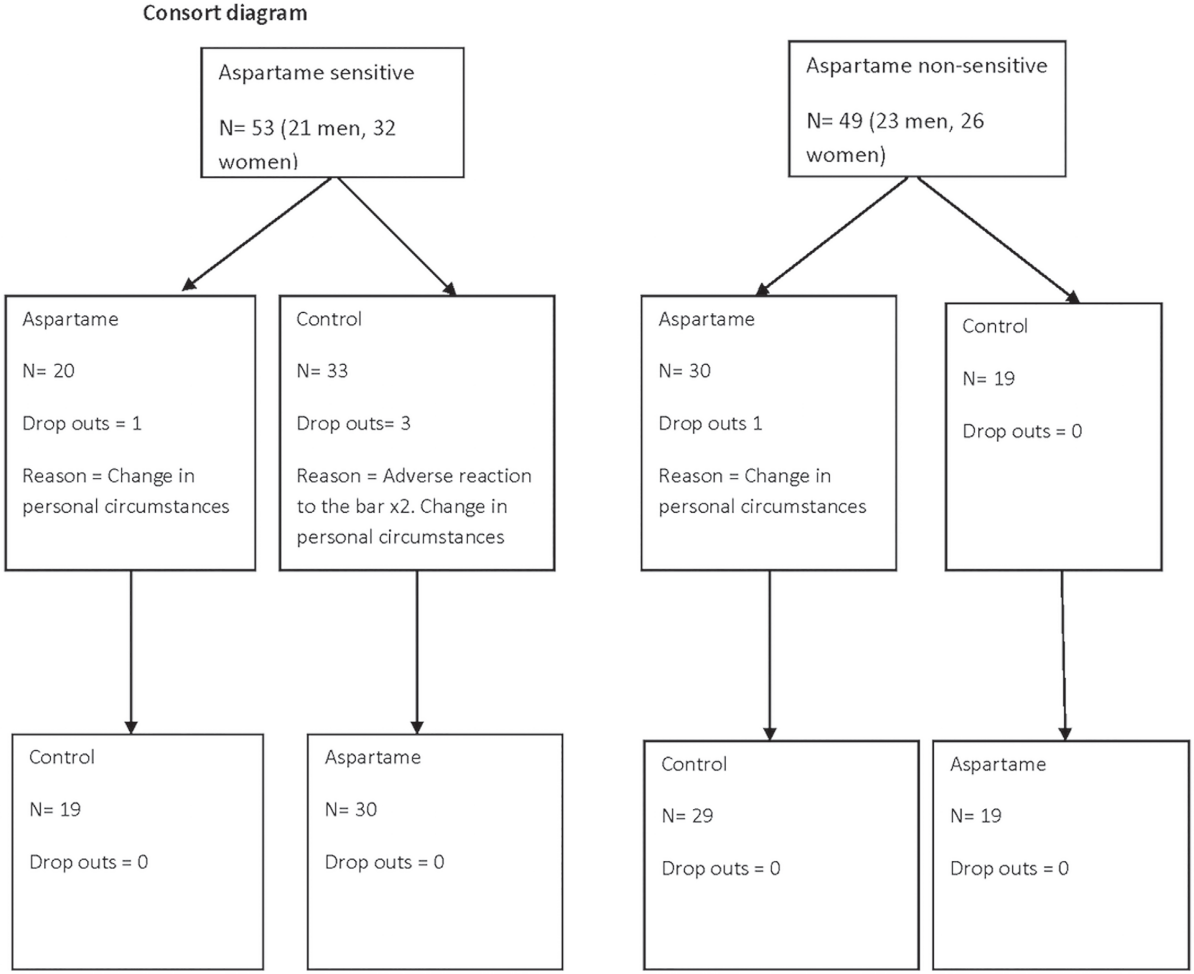
Consort diagram.

Although 268 self-reported aspartame sensitive individuals (AS) inquired, only 53 (21 men, 32 women) attended and gave their informed consent to take part in the study, with only 48 completing both sessions. Participants were matched by age and sex to 49 aspartame non-sensitive (NS) individuals (23 men, 26 women) (Table 1). Two participants (1 man, age 65 and 1 woman, age 70 years) from the aspartame sensitive group and one non-sensitive participant (1 man, age 41 years) dropped out due to changes in personal circumstances. One man was excluded because of a self-limiting gastro-intestinal upset during one session (revealed when unblinded to be after control). Consort diagram is given in Fig. 1.

**Table 1:** Baseline parameters before aspartame and placebo preparation in aspartame sensitive and control groups.

Parameter			
Group	Sensitive	Controls	p-value
Age (years)	50.53 ± 16.24	52.42± 15.38	0.56
Weight (kg)	88.28 ± 17.30	83.13 ± 20.34	0.19
Body mass index (kg/ m^2^)	30.14± 5.71	28.87 ± 5.9	0.29
Waist (cm)	97.03 ± 2.20	93.23 ± 2.57	0.27
Hip (cm)	106.27± 14.77	103.15 ±17.61	0.38

Data are means ± standard deviation

### Psychological results

**Baseline psychological state (Table 2)**. In MANOVA there were significant differences between sensitive participants and controls on: Perceived stress severity (F(1,43) = 6.13, p = 0.017); TAS identifying feelings (F(1,43) = 5.58, p = 0.023); TAS describing feelings (F(1,43) = 4.28, p = 0.045); TAS total score (F(1,43) = 5.70, p = 0.021). There was also a significant type of participant by order interaction for Perceived Stress Severity (F(1,43) = 5.64, p = 0.022). Preliminary analyses indicated that none of these variables explained variance in symptom rating, so final analyses excluded them.

**Table 2:** Aspartame sensitive and non-sensitive participants' scores on baseline psychological measures.

Groups		Anxiety (HADS)	Depression (BDI)	Depression (HADS)	Perceived Stress Severity	Life Events12m	Feelings (TAS)	Somatisation (Whiteley)	State Anxiety	Trait Anxiety
Sensitive	Mean	5.11	8.57	3.34	13.89	244.98	47.63	3.16	31.61	35.45
	N	47	37	47	46	50	46	45	41	42
	Std. Deviation	3.83	7.34	3.23	6.77	281.25	12.99	3.18	11.17	10.32
Control	Mean	4.86	5.43	2.30	11.07	279.04	43.16	2.63	29.83	32.28
	N	44	44	44	46	47	44	41	41	40
	Std. Deviation	3.74	6.40	2.46	6.00	457.95	11.60	2.57	9.53	9.80
Total	Mean	4.99	6.86	2.84	12.48	261.48	45.44	2.91	30.72	33.90
	N	91	81	91	92	97	90	86	82	82
	Std. Deviation	3.767	6.98	2.92	6.52	375.71	12.47	2.91	10.36	10.13

**Fig 2:**
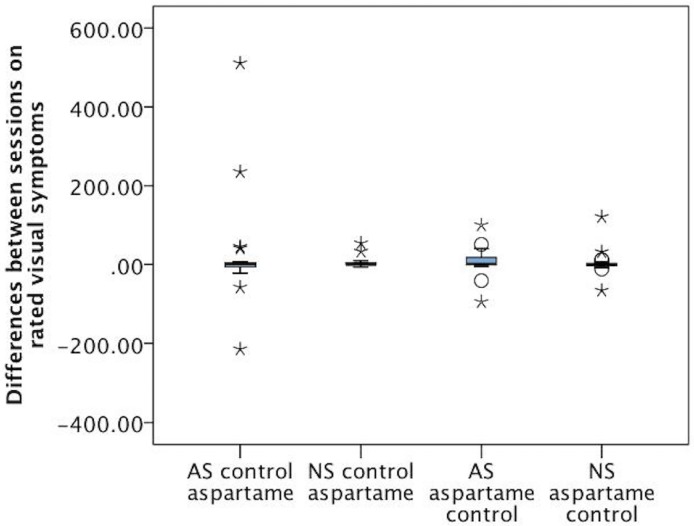
Differences between ratings of visual symptoms across session one and session two. Positive differences indicate that aspartame was rated higher than control. AS = Aspartame sensitive, NS = Non sensitive. It can be seen that there were no differences between groups on ratings, but the aspartame sensitive participants who rated control bars first included three outlier participants more extreme than any in the other groups.

**Visual analogue symptom scales over 4 hours of the trial**. There were small session by order interactions for: mood swings (F(1,85) = 6.26, p = 0.014); hotness (F(1,84) = 4.49, p = 0.037); arousal (F(1,85) = 5.16, p = 0.026; vision (F(1,85) = 5.62, p = 0.020). There were no session by order by type interactions. Post-hoc Mann Whitney U tests were conducted to compare aspartame and no aspartame bars in each session. There were no significant differences on any symptom. Visual inspection of the data suggested that differences in ANOVA were due to the continued non-normality of the symptom ratings, even after transformation. Fig. 2, shows box whisker plots for the differences between session one and session two ratings for visual symptoms, illustrating the absence of differences between groups and treatments, and the presence of outliers who rated a lot of symptoms in the sensitive group.

Main effects of participant type were found on total symptoms (F(1,77) = 10.57, p = 0.002 and nasal congestion (F(1,84) = 4.09, p = 0.046), indicating that sensitive participants tended to rate more symptoms to aspartame and control. Also, type by time interactions for headache (F(1,84) = 8.29, p = 0.014), nausea (F(1,85) = 11.65, p = 0.001), dizziness (F(1,85) = 11.17, p = 0.001), nasal congestion (F(1,84) = 7.14, p = 0.009, tingling (F(1,84) = 7.10, p = 0.009), thirst (F(1,82) = 4.11, p = 0.046), and bloating (F(1,75) = 6.69, p = 0.012), indicating that sensitive participants tended to rate more symptoms during the first test session.

### Biochemistry results

There were no differences in age (AS 50.5 ± 16.2 v NS 52.4 ± 15.4), weight (AS 88.3 ± 17.3 v NS 83.1 ± 20.3), BMI (AS 30.1 ± 5.7 v NS 28.9 ± 5.9), waist (AS 97.0 ± 2.2 v NS 93.2 ± 2.6), or hip circumference (AS 106.3 ± 14.8 v NS 103.2 ± 17.6), Baseline biochemical parameters were comparable in the AS and NS subjects; however, higher triglycerides (2.05 ± 1.44 vs. 1.26 ± 0.84mmol/L; p value 0.008) and lower HDL-C (1.16 ± 0.34 vs. 1.35 ± 0.54 mmol/L; p value 0.04) were observed in the AS group.

There was a significant increase in GLP-1 and a reduction in GIP, tyrosine and phenylalanine in both the aspartame sensitive and non-sensitive groups after aspartame and control preparations (Table 3). The changes in the above parameters were not significantly different between AS and NS groups and aspartame and control preparations.

**Table 3:** Changes in biochemical parameters after aspartame and control preparation in aspartame sensitive and non-sensitive group.

Parameter								
Group	Sensitive		Sensitive		Control		Control	
Treatment	Pre-aspartame	Post-aspartame	Pre-control	Post-control	Pre-aspartame	Post-aspartame	Pre-control	Post-control
	Mean ± SEM	Mean ± SEM	Mean ± SEM	Mean ± SEM	Mean ± SEM	Mean ± SEM	Mean ± SEM	Mean ± SEM
Glucose (mmol/L)	5.70 ± 0.24	6.19 ± 0.46	5.78 ± 0.31	6.32 ± 0.48	5.30 ± 0.21	5.39 ± 0.22	5.32 ± 0.23	5.51 ± 0.31
Insulin (μIU/mL)	15.02 ± 2.03	14.89 ± 2.14	14.10± 2.07	13.30 ± 2.05	10.26 ± 1.13	9.56 ± 1.23	10.74 ± 1.05	9.18 ± 1.17
HOMA_IR	3.70 ± 0.66	4.51 ± 1.15	3.90± 0.83	4.34 ± 1.12	2.54 ± 0.39	2.51 ± 0.47	2.59 ± 0.45	2.72 ± 0.65
TC (mmol/L)	4.79 ± 0.19	4.85 ± 0.19	4.68 ± 0.18	4.76 ± 0.18	4.67 ± 0.18	4.72 ± 0.18	4.65 ± 0.16	4.65 ± 0.17
Triglycerides (mmol/L)	1.98 ± 0.22	1.77 ± 0.16	1.86 ± 0.18	1.77 ± 0.18	1.28 ± 0.09	1.23 ± 0.09	1.25 ± 0.08	1.23 ± 0.09
LDL-C (mmol/L)	2.79 ± 0.16	2.87 ± 0.16	2.72± 0.15	2.76 ± 0.14	2.72 ± 0.13	2.78 ± 0.12	2.73 ± 0.10	2.73 ± 0.11
HDL-C (mmol/L)	1.16 ± 0.05	1.20 ± 0.05	1.16 ± 0.06	1.19 ± 0.06	1.35 ± 0.08	1.38 ± 0.08	1.35 ± 0.07	1.35 ± 0.07
GLP-1 pM	26.66 ± 1.83	57.85 ± 10.31**	30.00 ± 2.09	51.16 ± 8.76*	25.95 ± 1.38	59.67 ± 15.22	25.48 ± 1.28	45.82 ± 7.06**
GIP pg/mL	75.14 ± 3.82	48.28 ± 4.15***	75.95 ± 3.66	47.69 ± 3.06***	77.70 ± 4.19	49.19 ± 4.22***	76.98 ± 4.19	42.18 ± 2.77***
Tyrosine μM	76.04 ± 2.83	62.85 ± 2.90***	74.43 ± 3.09	62.20 ± 2.62***	69.85 ± 2.59	58.76 ± 2.23***	68.09 ± 2.62	57.01 ± 2.10***
Phenylalanine μM	82.01 ± 2.73	77.92 ± 2.48*	80.74 ± 2.55	74.80 ± 2.06***	76.21 ± 2.00	73.63 ± 1.86	77.85 ± 1.67	72.40 ± 1.87***
TP ratio	1.11 ± 0.03	1.29 ±0.04 ***	1.12 ± 0.04	1.26 ± 0.05***	1.13 ± 0.04	1.30 ± 0.04*	1.19 ± 0.04	1.32 ± 0.04***

To convert values for glucose to milligrams per deciliter, divide by 0.056.

To convert values for insulin to picomoles per liter, multiply by 6.

To convert values for cholesterol to milligrams per deciliter, divide by 0.0259.

To convert values for triglycerides to milligrams per deciliter, divide by 0.0113.

TC—Total cholesterol; LDL-C—LDL-cholesterol; HDL-C—HDL cholesterol; TG-Triglycerides

GLP-1—Glucagon-like peptide-1

GIP—Glucose-dependent insulinotropic polypeptide

HOMA—Homeostasis model of assessment—insulin resistance

TP ratio—Tyrosine/Phenylalanine ratio

Mean ± SEM—Mean ± Standard error of mean

AP difference—difference between aspartame and control group

P value –

* P < 0.05

** P < 0.01

*** for P < 0.001

### Metabonomics studies

**Fig 3:**
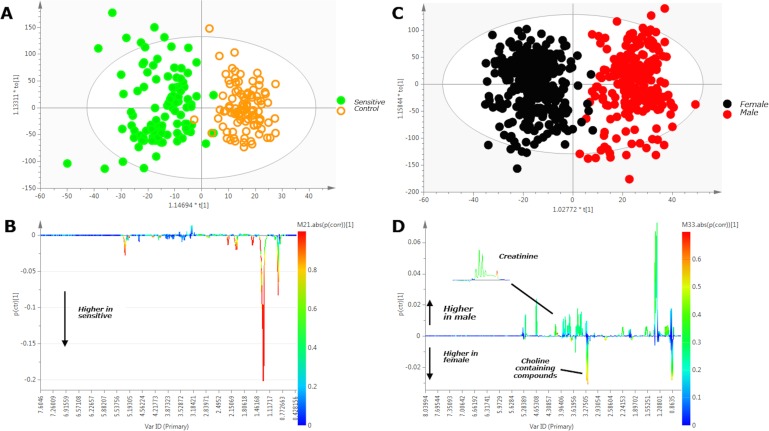
Analysis of 600 MHz ^1^H NMR human serum baseline (pre-bar consumption) data from sensitive and control participants (A), and female and male participants (C). 3A. OPLS-DA scores plot demonstrating that metabolic variation is dominated by inter-group (sensitive versus control) variation; 3B. OPLS-DA regression coefficient loadings plot (in the form of pseudo-NMR spectrum) of sensitive vs. non-sensitive confirming discriminatory peaks of interest identified by analysis of the raw spectra as being strongly associated with lipids. The signal orientation indicates a higher concentration of characteristic lipid metabolites in the sensitive population; 3C. OPLS-DA scores plot demonstrating clear separation of male (red symbols) from female (black symbols) subjects; 3D. OPLS-DA regression coefficient loadings plot (in the form of pseudo-NMR spectrum) male vs female confirming discriminatory peaks of interest identified by analysis of the raw spectra. The signal orientation indicates increased creatinine in males and increased choline-containing metabolites in females. For the loadings plots in B and D, the color-coded scale reflects the significance of the correlation of each metabolite with the separation (red indicates highest significance and blue indicates no significance).

The ^1^H NMR analysis indicated a metabolic difference in the blood serum samples attributed to variation in the serum lipid content between the two groups (AS vs NS). Fig. 3A and 3B, where the OPLS-DA scores plot along with the respective loadings plot in the form of a pseudo-NMR spectrum depicts these findings. Confirmation of the serum ^1^H NMR data was obtained using UPLC-MS lipidomics on a sample subset chosen randomly from each group (n = 12 per group). The sensitivity of this metabonomic approach was demonstrated as the models predicted the sex of individuals, irrespective of group (Fig. 3C and 3D).

No significant differences were found between the baseline samples from the AS and NS individuals using ^1^H NMR of the urine, nor were any significant differences found in the samples from the AS and NS individuals before or after aspartame or control bars.

## Discussion

This independent study set out to directly address the considerable anxiety surrounding aspartame consumption but found no difference in symptoms reported after aspartame and control, and no consequential differences between aspartame sensitive and non-sensitive participants; nor did consumption result in biochemical or metabolic differences. In combination, these findings assume that there is no acute adverse response to aspartame. Individuals reporting sensitivity to aspartame reported more symptoms, but after both bars equally and often during the first test session. These findings are in accord with the scientific data on aspartame [3,19,31].

There are a number of limitations. In order to minimise bias, participants were not monitored whilst completing rating scales, resulting in missing rating data. In addition, a small number of participants failed to complete the ratings at all. However, it is difficult to imagine a scenario where participants experiencing symptoms would leave relevant scales blank.

Regarding metabolic parameters, sensitive subjects had significantly lower HDL and higher triglyceride levels, reflected in the metabonomic data, suggesting that this was not a type 1 error. No other biochemical or metabonomic parameters differed between the two groups of participants.

There was a significant increase in plasma GLP-1 and reduction of plasma GIP-1 between baseline and 4 hour after both aspartame and control preparation in all study participants. GLP-1 usually rises one to four hours after nutrient intake. GIP levels are reported to peak between 15 and 45 minutes after meals, but there are no studies reporting GIP levels 4 hours after meals. The results suggest that the observed response of these incretin hormones with both the aspartame and control bar is due to the nutrients in the bar rather than aspartame.

There was a significant reduction of both phenylalanine and tyrosine 4 hours after ingestion of both aspartame and control bars. After oral ingestion, aspartame is rapidly hydrolysed within the lumen of the gastro-intestinal (GI) tract, with a half-life in the order of minutes[32] resulting in formation of methanol and the amino acids, aspartic acid and phenylalanine. Phenylalanine has a short half-life in plasma of approximately 1.7h[33] as such it was not expected that there would be any observable effects on blood phenylalanine levels under normal conditions, but the measure was undertaken so that any unusual metabolic effect should not go undetected. Methanol, whilst highly toxic, is subject to significant first pass metabolism via formaldehyde to formate. Also, it is known that formation of methanol from ingestion of aspartame is of lower magnitude than methanol from endogenous pathways and from other ingested sources, e.g. fruit juice [34–36]. As expected metabonomics failed to detect methanol or significant increases in formate in blood or urine as constitutive levels would mask any change.

It was anticipated that 48 individuals self-reporting aspartame sensitivity would be recruited within one year, but recruitment took 2.5 years despite high-level media coverage. This is exemplified by 147 individuals initially volunteering for the study before one aspartame sensitive individual participated; this likely reflects their genuine fear of aspartame consumption. Thus the achieved sample may not be representative of the population of people who believe they are aspartame sensitive, but it was impossible to recruit those most fearful.

This study looked only at acute effects and cannot exclude the possibility of chronic, cumulative effects of aspartame on biological parameters and on the psychological state[37,38]. Also, the dose given is smaller than the daily intake of many individuals. However, it is greater than the intake at which the people reporting aspartame sensitivity believe that they suffer symptoms[23].

## Conclusion

Measuring psychological condition, symptoms, biochemistry and metabonomics, there was no evidence of any acute adverse responses to aspartame. This independent study gives reassurance that the acute ingestion of aspartame does not have any detectable psychological or metabolic effects in humans.

## Supporting Information

S1 CONSORT Checklist CONSORT checklist. (DOC)

S1 Protocol Trial Protocol. (DOC)
