# Correction: Aspartame Sensitivity? A Double Blind Randomised Crossover Study

**DOI:** 10.1371/journal.pone.0126039

**Published:** 2015-05-07

**Authors:** The PLOS ONE Staff

The authors are listed out of order. The publisher apologizes for the error. Please view the correct author order, affiliations, and citation here:

Thozhukat Sathyapalan^1^, Natalie J. Thatcher^2¤^, Richard Hammersley^3^, Alan S. Rigby^4^, Fraser L Courts^1^, Alexandros Pechlivanis^5^, Nigel J. Gooderham^5^, Elaine Holmes^5^, Carel W. le Roux^6^, Stephen L. Atkin^7^

1 Academic Endocrinology, Diabetes and Metabolism, Hull York Medical School, University of Hull, Hull, United Kingdom,

2 Food Standards Agency, London, United Kingdom,

3 Department of Psychology, University of Hull, Hull, United Kingdom,

4 Department of Academic Cardiology, University of Hull, Hull, United Kingdom,

5 Faculty of Medicine, Imperial College, London, United Kingdom,

6 Diabetes Complications Research Centre, Conway Institute, University College Dublin, Belfield, Ireland,

7 Weill Cornell Medical College Qatar, Education City PO Box 24144, Doha, Qatar

Sathyapalan T, Thatcher NJ, Hammersley R, Rigby AS, Courts FL, Pechlivanis A, et al. (2015) Aspartame Sensitivity? A Double Blind Randomised Crossover Study. PLoS ONE 10(3): e0116212. doi:10.1371/journal.pone.0116212
