# Supplementary material for: Aspartame Sensitivity? A Double Blind Randomised Crossover Study
Source: PLoS One. 2015 Mar 18;10(3):e0116212. doi: 10.1371/journal.pone.0116212 (PMC4364783; doi:10.1371/journal.pone.0116212)
Supplement: S1 Protocol — (DOC) [file pone.0116212.s002.doc]

**Protocol S1**

**Full Title: Determination of the symptoms of aspartame in subjects who have reported symptoms in the past compared to controls: a pilot double blind placebo crossover study**

**Short title: Assessment of symptoms to aspartame of self diagnosed responders**

**Rec ref: 09/1304/46**

**Protocol details**

Version number and date: version 1.5 06/04/2011

EudraCT number: Aspartame is a food stuff and is under regulations of EFSA and FSA rather than MHRA

**Names (titles), roles and contact details of:**

Name, title, address and telephone number of the Chief/ Principal Investigator.

*Professor Stephen L Atkin*

*Head of Academic Diabetes, Endocrinology and Metabolism*

*Hull York Medical School*

*220-236 Anlaby Road*

*HULL, HU3 2RW*

*E-mail:* [*stephen.atkin@hyms.ac.uk*](mailto:stephen.atkin@hyms.ac.uk)

*Telephone: 01482 675365*

Name and address of sponsor:

*Hull and East Yorkshire Hospitals NHS Trust,*

*R & D department, Office 6, 2^nd^ Floor Daisy Building,*

*Castle Hill Hospital, Castle Rd,*

*Cottingham, East Yorkshire HU16 5JQ.*

Name and address of funder.

*Food Standards Agency*

*Aviation House*

*125 Kingsway London*

*WC2B 6NH*

Name, title, address and telephone number of statistician.

*Mr Alan Rigby*

*Department of Medicine*

*University of Hull*

*Department of Cardiology*

*Castle Hill Hospital, Castle Road*

*Cottingham, HU16 5JQ*

*Email: asr1960@hotmail.com*

Names and addresses of the clinical laboratories and other medical and/or technical departments and/or institutions involved in the study.

***Biochemical analysis will be undertaken in the clinical laboratories at Hull Royal Infirmary***

***Metabolomics will be undertaken by Imperial College, London***

**List of abbreviations and definitions**

**mg/kg bw – dose of aspartame in mg per kg bodyweight**

**EFSA – European Food Safety Authority**

**FSA – Food Standards Agency**

**Background information**

Prior research investigating the potential health effects of aspartame have been criticised by lobby groups as the work to date is not robust due to poor methodology or confounded as aspartame was not the main focus of the study. To prepare for a definitive study it is thought that a pilot study is necessary to prove the methodology, the preparation to be used and the acceptability of the study to the scientific community, food regulatory and other stakeholders. This will allow an extensive and comprehensive study to be powered appropriately.

This double blind pilot study aims to utilise a defined product, assess the efficacy of the necessary psychometric and biochemical methodologies to determine whether the perceived effects of aspartame can be detected in sufferers (of aspartame effects) compared to non suffers. This will allow the preparation of a full trial to be accurate in terms of endpoints studied, numbers of participants etc.

**Aims and Objectives**

To study the acute effects of aspartame on psychological symptoms, biochemistry and metabonomics.

To ascertain the optimal design for a larger study.

To validate that the product used for the trial is optimal and fit for purpose

To validate the questionnaire tools for the identification of symptoms of adverse events in individuals reporting self diagnosed adverse reactions.

To validate the biochemical assays in individuals reporting self diagnosed adverse reactions.

To determine the power needed for a large scale study for the investigation of subjects with self diagnosed adverse reactions to aspartame.

**Outcome:**

Confirm the robustness of the methodology for a definitive clinical trial.

**Study Design**

This is a double-blinded, placebo-controlled trial involving 50 self reported responders to aspartame (report that they have side effects when eating/ drinking products containing aspartame). Each participant will be given a single exposure to a bar containing aspartame as well as a matched placebo bar containing no aspartame. The dose of aspartame in the bars is 100mg, this is well below the Reference Daily Intake maximum recommended by EFSA of 40mg/kg body weight. In addition 25 participants with self-reported aspartame sensitivity and 25 matched control participants will be recruited to look at nocebo effect where they were given two lots of placebo (second part). The nocebo effect refers to the negative consequences of an inert treatment. The intention is to analyse the second part of the study only if any discriminating effect was observed between the two arms in the first part of the study.

*Plan for trial*

*Visit 1:*

Consent

Height, weight and waist measurements

Baseline bloods: full blood count and biochemical profile for safety

Baseline questionnaires of health and symptoms

- Beck depression inventory (BDI)
- Hospital Anxiety and Depression Scale (HADS)
- Trait anxiety perceived stress scale (STAI)
- Life events in last 5 years and 12 months (SRRS)
- Toronto Alexithymia scale
- Whiteley Index of Hypochondriasis

*Visit 2:*

Attend fasting

Fasting bloods: insulin, glucose, GLP1, GIP, biochemical profile and glucose

Give test or placebo challenge snack bar

Use of Visual Analogue Scales to assess effects of the aspartame/ placebo every 15 minutes for 2 hours, then at 3 hours and at 4 hours

Repeat bloods 4 hours following the consumption of the bar: insulin, glucose, GLP1, GIP, biochemical profile and glucose.

After lunch and recording of any adverse events or symptoms, subjects go home.

Telephone call 48 hours after visit to remind about questionnaires and monitor any side effects

One week between visits.

*Visit 3:*

Attend fasting

Fasting bloods: insulin, glucose, GLP1, GIP, biochemical profile and glucose

Give test or placebo challenge snack bar

Use of Visual Analogue Scales to assess effects of the aspartame/ placebo every 15 minutes for 2 hours then at 3 hours and at 4 hours

Repeat bloods 4 hours following the consumption of the bar: insulin, glucose, GLP1, GIP, biochemical profile and glucose.

Urine to be collected at the start of the study, at the end of the study (4 hours). If willing to do so, a urine sample will be requested at 12 hours and 24 hours: prepaid containers will be provided to post these back to the centre.

Imperial College, London will be subcontracted to measure aspartame and its breakdown products in blood and urine by the use of metabolite analysis using NMR and mass spectrometry. Samples will be sent to them in an anonymous manner to preserve confidentiality.

After lunch and recording of any adverse events or symptoms, subjects go home.

Telephone call 48 hours after visit to remind about questionnaires and monitor any side effects

*Criteria for discontinuation of individual subjects, parts of the study or the entire study*.

As it is a relatively short intervention with a well tolerated food stuff this is felt to be incredibly unlikely.

*Individuals may be withdrawn if:*

- If they lose capacity
- If they develop an allergy or adverse reaction to the aspartame or the ingredients in the bar
- If unable to eat less than 75% of the snack bar

For the part or the whole study to be withdrawn

- If the Food Standards Agency said that aspartame was unsafe as a food ingredient.

Plan for the Study

| Procedure | Visit 1 | Visit 2 | Visit 3 |
| --- | --- | --- | --- |
|  | Week 0 | Week2 | Week3 |
| Consent | X |  |  |
| Blood tests –   - full blood count - biochemical profile | X |  |  |
| - Baseline Questionnaires - Beck depression inventory (BDI) - Hospital Anxiety and Depression Scale (HADS) - Trait anxiety perceived stress scale (STAI) - Life events in last 5 years and 12 months (SRRS) - Toronto Alexithymia scale - Whiteley Index of Hypochondriasis | X |  |  |
| Height, weight and waist measurement | X |  |  |
| Fasting blood test-  Insulin, glucose, GLP1, GIP, biochemical profile and glucose |  | X | X |
| Feeding of snack bar, with or without aspartame |  | X | X |
| Completion of Visual Analogue Scale every 15 minutes for 2 hours |  | X | X |
| Blood test 4 hours after eating bar:  Insulin, glucose, GLP1, GIP, biochemical profile and glucose |  | X | X |
| Urine aliquot at baseline, 4, 12 and 24 hours after eating bar, |  | x | x |
| Giving of Speilburger State trait anxiety questionnaire and food symptom log |  | X | X |
| Reminder telephone call and review of symptoms, 48 hours after the test | X | X | X |

A schematic diagram of the trial design, procedures and stages (can be in a form of a table).

**Primary Endpoints**

**This is pilot study**

**The primary endpoint is the testing of the rigour of the study methods and design.** To determine the power needed for a large scale study for the investigation of subjects with self diagnosed adverse reactions to aspartame.

To study the acute effects of aspartame on psychological symptoms, biochemistry and metabonomics.

**Secondary Endpoints**

To validate that the product used for the trial is optimal and fit for purpose

To ascertain the optimal design for the main study.

To validate the questionnaire tools for the identification of symptoms of adverse events in individuals reporting self diagnosed adverse reactions.

To validate the biochemical assays in individuals reporting self diagnosed adverse reactions.

**Subject selection**

This study has 2 groups of subjects, those who report having symptoms when consuming aspartame and those who consume aspartame with no ill effects.

The study will recruit 50 subjects in each group.

The responders will be approached by the lobby and interest groups who have accumulated a list of individuals who have an interest in aspartame and report side effects when consuming it. Information will be provided to these groups and a letter with a summary information sheet will be provided to these group to distribute amongst their members. Then the individuals who are interested in taking part in the study will contact the research department to arrange an appointment.

The healthy volunteers will be recruited via a poster and they will be encouraged to contact the research department for study information. The poster will be displayed in primary care centres, university buildings and within the hospital trust. Following subjects contacting the research centre, information will be sent out, following the receipt of the information subjects will be allowed to read the information and asked if interested in further participation in the study, if so a screening appointment will be arranged.

Number of centres involved:

One NHS site: Hull and East Yorkshire Hospitals NHS Trust

One Academic site: University of Hull

Over 1000 side effect reports have been collated by interest groups in the UK, therefore about 100 are likely to be from the Yorkshire region, therefore before requiring subjects to travel excessive distances about 50% of the local individuals who respond to aspartame will need to be recruited. The recruitment of healthy volunteers is not foreseen to be a problem as the organisations who will be displaying the posters employ over 10,000 people and have even greater numbers accessing their buildings every month.

**Inclusion criteria:**

***Healthy volunteers***

No known allergies

No concomitant medical condition

No medication

***Aspartame reactors***

Reported effect of aspartame on ingestion

No allergies to other food substances

**Exclusion criteria:**

***Healthy volunteers***

Allergies to food or medication

A concomitant medical condition

Prescription medication

Refusal for GP to be informed

***Aspartame reactors***

Allergies to food or medication

A concomitant medical condition

Prescription medication

Refusal for GP to be informed

**Subject recruitment**

Recruitment process: Through the 2 routes previously identified

- Payment of participants.

Subjects will be given reasonable travel and parking expenses. Subjects will also be provided with lunch or a voucher to cover their lunch if attending the centre fasting.

- Details of procedures, tests, screening carried out to assess trial suitability.

Screening will be via interview and symptom review with a study doctor

- Provision of patient information sheet (see appendix 1).
- Gaining patient consent

Consent will be obtained by a trained member of the research team. This will be taken after a full history has been taken by a study doctor to investigate the potential risk to the subject and suitability for the study. As the study involves a number of validated questionnaires which are in English, it is important that English is the subject’s first language as interpretation may be effected if English is a second language.

- Detail of enrolment procedure.

Subjects having contacted the centre following initial information being sent to them as previously described, they will have the nature of the study explained to them prior to having information and nature of the study explained to them. Then the subjects will be invited to attend for consent and screening visit.

**Randomisation**

The randomisation will be performed by Campden BRI, UK. A computer generated randomisation list will be used to provide balanced blocks of patient numbers for each of the two treatment groups. A one to one treatment allocation will be used. The block size will not be revealed.

**Blinding and other measures taken to avoid bias**

The study will be double blind, with the active being snack bar containing 100mg aspartame and the placebo being an identical bar containing no aspartame. Blinding will be done at manufacture and the code will be placed in a sealed envelope only to be opened in emergency.

**Subject compliance**

Subjects will need to eat at least 75% of the bar.

Subjects will be reviewed by the study team 48 hours after their withdrawl to monitor any adverse events.

**Withdrawal of Subjects**

**If a subject is withdrawn, follow up by telephone with the study team 48 hours, to review any adverse events. Where possible they will be replaced in the study.**

**Treatment of subjects**

The treatment will be with 2 snack bars given individually on 2 separate days 1 week apart, 1 bar containing 100mg of aspartame the other being aspartame free. Other ingredients include spices, crisped rice and dried fruit.

**General information**

Snack bar containing aspartame (or aspartame free)

**Use of treatment within the trial**

The snack bar is to be eaten by fasting subjects, subjects will be asked to eat 2 bars given in a double blinded and randomised order, 1 containing aspartame and 1 is aspartame free. These bars will be given to the subjects 1 week apart

Subjects will be eating the bars in the centre and study staff will check that at least 75% of the bar is eaten.

Each bar contains either no or 100mg aspartame

A dispensing / compliance log will be kept with the case report form.

Bars will be made in accordance with Good Manufacturing Practice*.* Shelf life will be adequate for the duration of the trial

The bar will not necessarily be available for commercial use following the trial, a registered dietitian will be available to participants to discuss the role of aspartame and artificial sweeteners in their diet.

It is important that the participants are not taking regular medication

No changes in medications are permitted during the trial except general over the counter treatments, e.g. painkillers

**Efficacy Assessments**

**Efficacy will be measured by insulin, glucose, gut hormones and inflammatory markers.**

**All samples will be stored and batched for analysis at the end of the study.**

**As detailed above** Imperial College, London will be subcontracted to measure aspartame and its breakdown products in blood and urine by the use of metabolite analysis using NMR and mass spectrometry. Samples will be sent to them in an anonymous manner to preserve confidentiality.

**Safety Assessments**

This is a safety study, assessing the effect of aspartame will be by questionnaires and symptom diaries along with a review of the biochemical results.

These will be analysed at the end of the study, as these effects are likely to be small.

No serious adverse events are expected in the trial.

Detail the procedures that will be followed in the event of adverse events in the trial. *Adverse events will be reported in accordance with HEYHT R & D department adverse event reporting procedures. Serious adverse events will be notified to HEYHT R & D dept. within 24hrs of investigators becoming aware of the event using the SAE/SUSAR initial and follow-up report forms provided by R & D. All adverse events (serious and non-serious) will be recorded in patients data collection forms (CRFs) using R & D’s adverse event report form. All adverse events will be recorded in patients medical records.*

All subjects following an adverse event will be followed by the research team 4 weeks following the reported event.

**Data collection**

Data will be collated in patient notes and a case report form in line with trust guidelines and good clinical practice.

This study consists of 3 visits, blood samples and questionnaires will be used to collect data at each visit.

Data will be collected by the study team, this will include a research nurse, dietitian and medical registrar. Data will be anonymised, with only be identified by a study number and the study identification.

Provide a detailed list of all data (outcome variables, explanatory variables etc) to be collected, with each description including:

**Sample Size**

The ability to estimate an appropriate sample size for the pilot is not possible as there are no comparable studies for guidance. This pilot will allow the main study to be powered appropriately on a specific factor. However, Power and sample size for pilot studies has been reviewed by Birkett and Day (Birkett MA, Day SJ. Internal pilot studies for estimating sample size. Statistics in Medicine 1994; 13:2455-2263). They concluded that a minimum of 20 degrees-of-freedom was required to estimate effect size and variability. Hence, we intend to recruit 50 patients per group (75-105 maximum) allowing for drop-outs and covariate adjustment.

.

**Statistical Analysis**

Data will be paired, tested for normality, if normally distributed will be analysed by paired t-test with 2 tails and non-parametric data will be analysed using Wilcoxon-signed rank testing. ANOVA will be used to account for any differences in the population e.g. smoking.

**Quality control and quality assurance**

**Monitoring**

Arrangements for monitoring/auditing conduct of the research. *The study will be monitored in accordance with HEYHT R & D department’s standard operating procedures to ensure compliance with UK GCP regulations. All trial related documents will be made available upon request for monitoring by R & D monitors and for inspection by the MHRA.*

The research will be reviewed by the Food Standards Agency as part of the contract arrangements.

**Ethical considerations**

The considerations for this study include that the subjects who have reported symptoms following aspartame, they may wish to participate however this may not be in their best interest either in terms of physical or mental health. This will be discussed with the study team including a research dietitian and doctor.

The questionnaire will investigate a range of psychological traits including anxiety and hypochondria, this will be explained to potential subjects. Also clinical supervision will be available for the research team in delivering these questionnaires and psychological support will be available for subjects if it found to be necessary.

**Ethics, MHRA and R&D approval**

*The study will be performed subject to Hull and East Riding Local Research Ethics Committee favourable opinion, Site Specific Assessment (SSA) approval and HEY Trust R & D approval.*

**Research Governance**

*This study will be conducted in line with the International Conference for Harmonisation of Good Clinical Practice (ICH GCP) guidelines; and the Research Governance Framework for Health and Social Care.*

**Data handling and record keeping**

Professor Atkin will act as the data custodian and is responsible for the storage, handling and quality of the study data

Utilising the trusts *IT Services Department facility which has a backup procedure approved by auditors for disaster recovery. Servers are backed up to tape media each night. The tapes run on a 4 week cycle. Files stay on the server unless deleted by accident or deliberately. Anything deleted more than 4 weeks previously is therefore lost*. *Additional ‘archive’ backups are taken for archived data, so data should not be lost from this type of system e.g. FileVision which stores Medical Records. Tapes are stored in a fireproof safe.*

Data will be collected in the case notes and in the case report form to allow for cross referencing to check validity

*Study documents (paper and electronic) will be retained in a secure (kept locked when not in use) location during and after the trial has finished. All essential documents including source documents will be retained for a minimum period of 15 years after study completion (last patient, last visit). A label stating the date after which the documents can be destroyed will be placed on the inside front cover of the case notes of trial participants.*

*Data will be collected and retained in accordance with the Data Protection Act 1998.*

**Finance**

Provide any details of the financial arrangements for the study if not assessed in a different document.

This study has been funded by the Food Standards Agency

Provide details of any payments to be made to participants.

Participants will be paid reasonable travel and parking expenses. For the 2 visits which are longer and require subjects to attend fasting provisions will be made for lunch, either by supplying it directly or by providing a voucher.

**Indemnity**

Arrangements for providing cover for non-negligent and negligent harm.

*This is an NHS-sponsored research study. If there is negligent harm during the clinical trial when the NHS body owes a duty of care to the person harmed, NHS indemnity covers NHS staff and medical academic staff with honorary contracts only when the trial has been approved by the Trust R & D department. NHS indemnity does not offer no-fault compensation and is unable to agree in advance to pay compensation for non-negligent harm.*

**Reporting and dissemination**

This study will be reported at national and international conferences along with a number of papers in peer reviewed academic journals. A review of the study will also be made available to participants. Reporting of the study findings will be in accordance with the CONSORT statement (Consolidated Standards of Reporting Trials <http://www.consort-statement.org/>).

**Signature page**

Chief/ Principal Investigator

Professor Stephen Atkin

Signed _______________________________ Date_________________________

Sponsor, on behalf of Hull And East Yorkshire Hospitals NHS Trust

Name ________________________________

Signed _______________________________ Date ________________________

Funder, on behalf of the Food Standards Agency

Name _________________________________

Signed ________________________________ Date ________________________

*Adapted with kind permission from SUHT R & D Guide to writing a protocol for a CTIMP and from UBHT R & D Information Sheet No.1a, v1.0 10-11-04.*
